# The correlation between depression and haemodialysis adequacy in chronic kidney disease patients in South Africa

**DOI:** 10.4102/hsag.v31i0.3275

**Published:** 2026-08-31

**Authors:** Bongani V. Miya, Ntagi G. Mariri, Chevon Clark

**Affiliations:** 1 Department of Health Sciences, Faculty of Health and Environmental Sciences, Central University of Technology, Bloemfontein, South Africa

**Keywords:** chronic kidney disease, haemodialysis, mental health, depression, dialysis adequacy, beck’s depression inventory, mortality

## Abstract

**Background:**

Chronic kidney disease (CKD) is a global health concern projected to be the fifth most prevalent chronic disease by 2040, associated with high mortality and significant psychosocial burdens.

**Aim:**

This study aimed to explore and describe the correlation between depression and haemodialysis adequacy in CKD patients.

**Setting:**

Research was conducted across five units in the Free State and Northern Cape provinces, South Africa.

**Methods:**

This quantitative, cross-sectional, prospective analytic study included 78 patients selected via convenience sampling, and trustworthiness and ethical principles were adhered to throughout the study. A biostatistician analysed the raw data using the statistical analysis system (SAS). Data collected over 3 months involved objective measurements of online dialysis adequacy (Kt/V) and pathology (creatinine, urea), alongside subjective depression assessments using the Beck Depression Inventory (BDI).

**Results:**

The median BDI score was 17 (Interquartile range [IQR]: 13–22), reflecting mild-to-severe depressive symptoms. The median online Kt/V was 1.04. A significant inverse association was found between BDI scores and Kt/V (*p* = 0.0231), indicating that higher depressive symptoms correlate with lower adequacy. No significant relationships were observed between depression and creatinine or urea levels.

**Conclusion:**

Lower dialysis adequacy is significantly associated with increased depressive symptoms. Findings highlight the urgent need to integrate mental health screening and psychosocial support into routine renal care to improve patient outcomes.

**Contribution:**

The findings highlight the necessity of psychosocial support, patient compliance, and education to improve employment and nutritional security, and advocate for comprehensive medical insurance coverage.

## Introduction

Chronic kidney disease (CKD), which is projected to rank fifth in prevalence among chronic diseases by 2040, is defined as kidney structure or function abnormalities persisting for at least 3 months, often with an estimated glomerular filtration rate below 60 mL/min/1.73 m^2^ (Aghsaeifard et al. 2022; Bahall, Legall & Lalla 2023). Chronic kidney disease was responsible for 74% of global deaths in 2019, and by 2023, CKD-related mortality rates in South Africa had increased by 67% (Bohm et al. 2024; Hariparshad et al. 2023). Among the factors contributing to poor outcomes, depression stands out as a critical predictor. It is associated with lower quality of life, increased hospitalisations and higher mortality rates (Chen & Tsai 2010). In 2021, the estimated national prevalence of depression in South Africa ranged from 14.7% to 38.8% (Shezi et al. 2024). Despite its prevalence, depression in dialysis patients is mostly undiagnosed, impeding optimal patient care (Ngema & Ramalepa 2025).

Haemodialysis is the main form of kidney replacement therapy in CKD patients in sub-Saharan Africa (Cedeno et al. 2020). Despite the therapeutic advances in chronic dialysis, the mortality rate among patients on haemodialysis is eight times higher than for the general population (Eynde et al. 2022).

Haemodialysis is also associated with a high prevalence of psychological problems, which are believed to increase the morbidity and mortality rates in haemodialysis patients (Gela et al. 2024; Halili et al. 2021). The risk of death in CKD patients with mental disorders ranges from 11% to 66%, and the risk of hospitalisation can reach up to 90%, compared to CKD patients without common mental disorders (Hariparshad et al. 2023).

Dialysis adequacy is a critical aspect of haemodialysis care and has been shown to influence survival among patients receiving maintenance haemodialysis. It encompasses not only the effectiveness of solute clearance but also the maintenance of patients’ overall health, mental well-being, and biochemical balance, thereby improving quality of life and reducing morbidity and mortality (Aghsaeifard et al., 2022). Two methods of calculating Kt/V are commonly used: the Daugirdas formula and online clearance monitoring (OCM). The current study focused solely on the OCM-based Kt/V calculations. The quality of life with end-stage kidney disease (ESKD) on maintenance haemodialysis depends on consistent and efficient removal of uremic toxins (Pahari & Kumar 2023). While maintenance haemodialysis serves as the main therapy for patients with ESKD, it is also associated with a high prevalence of psychological problems such as depression and anxiety (Mosleh et al. 2020). Depression is three to four times more prevalent in CKD and ESKD patients compared to the general population and occurs at higher rates than in individuals with other chronic illnesses, such as diabetes (12% – 18%), coronary artery disease (15% – 23%), and chronic obstructive pulmonary disease (around 25%) (Shirazian et al. 2016).

In dialysis centres, where access to psychiatric care is often restricted, diagnostic scales are routinely employed to screen for depression in patients with ESKD. The Beck Depression Inventory (BDI) is a widely used self-report tool for screening and measuring the severity of depression (Hajduska-Der et al. 2022). The BDI comprises 21 questions assessing symptoms and attitudes related to depression, with each item rated on a scale from 0 to 3. Its versatility allows it to be administered in both clinical and non-clinical settings. Scores range from normal mood variations to extreme depression, providing a reliable measure for monitoring symptom progression and treatment efficacy (Hajduska-Der et al. 2022). Since its development in 1961, the BDI has been widely adopted worldwide for assessing depression not only in clinical patients but also in the general population (Hassan, Said & Ibrahim 2022). This study explores the correlation between depression and haemodialysis adequacy in CKD patients in the Northern Cape and Free State provinces of the Republic of South Africa.

## Research methods and design

### Study setting

This study was conducted across five private healthcare centres in the cities of Bloemfontein, Kroonstad, and Kimberley, in the Republic of South Africa. The private facilities in the Free State province included Pelonomi, Universitas, Bram Fischer, and Kroonstad. In the Northern Cape province, the private study site was Kimberley. Of the 120 patients seen at these facilities, only 78 met the inclusion criteria for the study. The study was conducted only in private settings, as public hospitals did not grant approval, citing concerns about research burden on dialysis patients and prioritising internal staff research projects.

### Study design

This study adopted a quantitative, cross-sectional, prospective analytic research approach to explore the correlation between depression levels and dialysis adequacy in CKD patients. This design was chosen because it enables observation and analysis of relationships between variables over time, ensuring a robust understanding of the interplay between mental health and dialysis outcomes (American Journal Experts 2022). The study is more accurately described as a quantitative cross-sectional analytic study with prospective data collection. The cross-sectional component refers to the measurement of both depression and haemodialysis adequacy at a single point in time for each participant, which is appropriate for assessing their correlation, as cross-sectional studies provide a snapshot of variables within a population at a specific time (Thomas 2023). The prospective aspect relates to the method of data collection, whereby respondents are recruited and assessed in real time as they present for haemodialysis, rather than relying on retrospective records. Thus, the study does not involve follow-up over time but prospectively collects cross-sectional data, ensuring standardised measurement and improved data quality, as cross-sectional studies involve the simultaneous measurement of exposure and outcome without longitudinal follow-up (Prabhakar & Kaushal 2024).

### Study population and sampling

In determining an appropriate sample size, we considered the total number of patients at each study site and the study’s inclusion and exclusion criteria. A total population sampling approach was employed.

This approach was suitable given the small, well-defined, and accessible population, reducing sampling error.

However, selection bias is acknowledged, as findings from private clinics may not be fully generalisable to public-sector patients due to differences in socioeconomic status, access to care, and health system resources.

The study evaluated the entire accessible population of 120 patients across five centres. Following the systematic application of inclusion and exclusion criteria, all 78 eligible individuals were included in the study to ensure a homogeneous group and maximise statistical power, as confirmed through consultation with a biostatistician. This approach aligned with recommendations that sampling strategies in quantitative research should closely match the study population and objectives to ensure the validity and reliability of findings (Creswell 2023).

#### Inclusion criteria

- Chronic haemodialysis respondents aged 18 to 65 years.
- Respondents undergoing nocturnal dialysis.
- Patients who provided informed consent.

#### Exclusion criteria

- Paediatric patients.
- Pregnant respondents.
- Holiday patients (those visiting centres for a few haemodialysis sessions while on holiday).

### Data collection

Respondents were recruited from the selected healthcare centres through collaboration with healthcare professionals and patient care teams. Recruitment strategies included direct engagement with potential respondents and the distribution of study information sheets. The principal investigator introduced the study’s concept to all patients at each location, encouraging both patients and nursing staff to actively participate and freely express their views and questions relating to the study. After satisfactorily addressing all inquiries, the study team, in partnership with the nursing staff, identified patients who expressed interest in participating in the study.

The inclusion and exclusion criteria were explained in detail to the willing respondents to ensure clarity and understanding. Subsequently, an informed consent form and a study information sheet were distributed to the willing respondents. After the consultation sessions concluded and respondents expressed their satisfaction, they signed a consent form.

Data collection was conducted over a 3-month period (June 2024 to August 2024). Respondents attended three haemodialysis sessions per week, resulting in a total of 12 sessions per month and 36 sessions over the study period.

Measurement of dialysis adequacy:

- Kt/V calculations: Automatically computed by the haemodialysis machine during each session.
- Blood tests: Blood samples were collected once a month, either on the first or last dialysis day, to assess clearance levels.

Depression assessment: Respondents completed the BDI questionnaire at the start of the study. The questionnaire was administered once and analysed in consultation with a qualified psychologist.

#### Demographic data

Demographic data collected included age, sex, employment status, level of education, and marital status.

These variables were used to describe respondents’ socio-demographic characteristics, assess their comprehension of the study questionnaire, and identify potential social support structures. The information was obtained directly from respondents at the beginning of the study across all five centres.

#### Clinical data

The BDI questionnaire comprises 21 questions assessing symptoms and attitudes related to depression, with each item rated on a scale from 0 to 3. Scores range from normal mood variations to extreme depression, providing a reliable measure for monitoring symptom progression and treatment efficacy (Hajduska-Der et al. 2022). Since its development in 1961, the BDI has been widely adopted worldwide for assessing depression not only in clinical patients but also in the general population (Hassan et al. 2022). Clinical data, such as the underlying cause of CKD, simultaneous presence of other medical conditions, currently prescribed medications, complications arising from treatment, and duration of dialysis, were obtained from the respondents’ medical records. The BDI questionnaire was completed by all 78 respondents at the study’s outset, with scores reflecting the level of depression.

Clear ethical procedures were in place for psychological support and referral: respondents who experienced emotional discomfort during questionnaire completion were given the option to pause or discontinue the assessment by the psychologist, who was part of the study team, and provided immediate support. Where necessary, respondents were referred to appropriate mental health services within the local healthcare system for further assessment and care. These procedures ensured participant safety, psychological well-being, and adherence to ethical requirements for managing distress in mental health-related research.

### Data analysis

This study employed statistical analysis system (SAS) software (Cary, NC, United States) to analyse the collected data. Data were captured in a Microsoft Excel spreadsheet (Redmond, WA, United States) by the principal investigator, screened for completeness, cleaned for missing values, and quality checked before submission to a biostatistician for analysis. The Shapiro–Wilk test was used to assess the normality of numerical variables. Categorical variables were summarised using frequencies and percentages, and group comparisons were performed using the chi-square test. Normally distributed continuous variables were compared using Student’s *t*-test and analysis of variance (ANOVA), while non-normally distributed variables were analysed using the Mann–Whitney U test and Kruskal–Wallis test. Chicco, Sichenze and Jurman (2025) explain that statistics provides researchers with a range of valuable analytical tools, including several univariate tests designed to examine relationships between numerical variables. These include Student’s *t*-test, Mann–Whitney U test, chi-squared test, and Kruskal–Wallis test. The authors further note that such tests yield various statistical outputs, particularly *p*-values, which quantify the strength of evidence against the null hypothesis and inform decisions to either reject or accept it based on a predetermined significance level. Correlation analysis and logistic regression were conducted to examine the association between mental health and haemodialysis adequacy. A significance level of α = 0.05 was applied to all statistical analyses to address the study objectives comprehensively.

### Validity and reliability

The study ensured validity and reliability through several methodological strategies. Firstly, rigorous inclusion and exclusion criteria were applied to ensure a well-defined and homogeneous study population, thereby enhancing internal validity and reducing selection bias. Secondly, standardised and validated measurement tools were used, including the BDI tool for assessing depressive symptoms and Kt/V calculations for determining haemodialysis adequacy, both of which are widely recognised for their reliability and clinical validity. In addition, data collection procedures were standardised across all respondents to ensure consistency in measurement and reduce inter-observer variability. Furthermore, appropriate statistical techniques were employed to enhance the accuracy, reliability, and reproducibility of the findings, including methods to control for potential confounding variables where applicable. Collectively, these measures strengthened the scientific rigour of the study and ensured that the findings are both credible and generalisable within the study context.

### Ethical considerations

Ethical considerations were of the utmost importance in this study. Ethical clearance was obtained from the University of the Free State Health Sciences Research Ethics Committee (ethical clearance number: UFS-HSD2023/2414/3004). Additionally, ethical approval for the study was obtained from the private company’s research committee prior to the commencement of the research. Respondents received an information sheet detailing the study’s objectives, expectations, duration, and investigator contact information. Informed consent was obtained, and respondents were informed of their right to withdraw from the study at any time without facing negative consequences.

Pseudonymised data were used to protect respondents’ confidentiality. De-identification techniques, such as pseudonymisation and anonymisation, play an important role in facilitating such secondary uses and disclosures of data (Thaldar 2023). In this study, data were securely stored in the university’s digital storage system, with physical documents, including questionnaires and chronic dialysis sheets, locked in a safe.

Portable devices used for data collection were encrypted, and hard copies containing identifiable information were securely stored and accessible only to authorised research team members. An information sheet detailing the study’s objectives, expectations, duration, and the investigator’s contact information was provided to each respondent. All willing respondents gave informed consent and were then informed that they had the right to withdraw from the study at any time without facing negative consequences. No respondents withdrew from the study.

## Results

### Demographic results

Of the 78 respondents in this study, 45 were female, and 33 were male. The mean age of the respondents was 50 years. Ten respondents used wheelchairs, and one was visually impaired and was assisted by nursing staff to complete the questionnaire; the nursing staff read the questions and allowed the respondent to choose the answer. All 78 respondents had some level of formal education.

### Dialysis adequacy assessment

Dialysis adequacy was evaluated using daily and average monthly online Kt/V values, calculated from clinical data collected during the study period; the findings are summarised in Table 1. It presents the distribution of key clinical variables, such as Kt/V values, using medians and interquartile ranges, which are appropriate for skewed biomedical data. These measures provide a baseline understanding of variability in dialysis adequacy, supporting accurate interpretation of treatment effectiveness and the identification of clinically meaningful differences among respondents.

**TABLE 1:** The median test for overall Kt/V (*N* = 78).

Variable	Median	25th Pctl	75th Pctl	Min	Max
Month 1 Kt/V average	1.03	0.99	1.11	**0.68**	**1.24**
Month 2 Kt/V average	1.03	0.98	1.08	0.70	**1.76**
Month 3 Kt/V average	1.07	0.98	1.12	0.83	1.59
Overall, Kt/V average	**1.04**	**1.00**	**1.10**	0.76	**1.34**

Note: Bold-faceted values indicate significant value.

Pctl, interquartile range percentile; Min, minimum; Max, maximum.

The average Kt/V for month 1 and month 2 remained consistent, with a median of 1.03. However, the month 2 recorded a maximum Kt/V value of 1.76, compared with the month 1 maximum Kt/V of 1.24 and a minimum of 0.68. The overall median Kt/V over the 3 months was 1.04, with a maximum Kt/V value of 1.34 and an interquartile range of 1.00 to 1.10. This indicates that Kt/V values remained stable over the 3 months, with an overall median of 1.04, below the minimum prescribed target of 1.2. Most patients, therefore, did not achieve the recommended dialysis adequacy, although a few individuals reached higher Kt/V values, as reflected in the maximum measurements.

**FIGURE 1:**
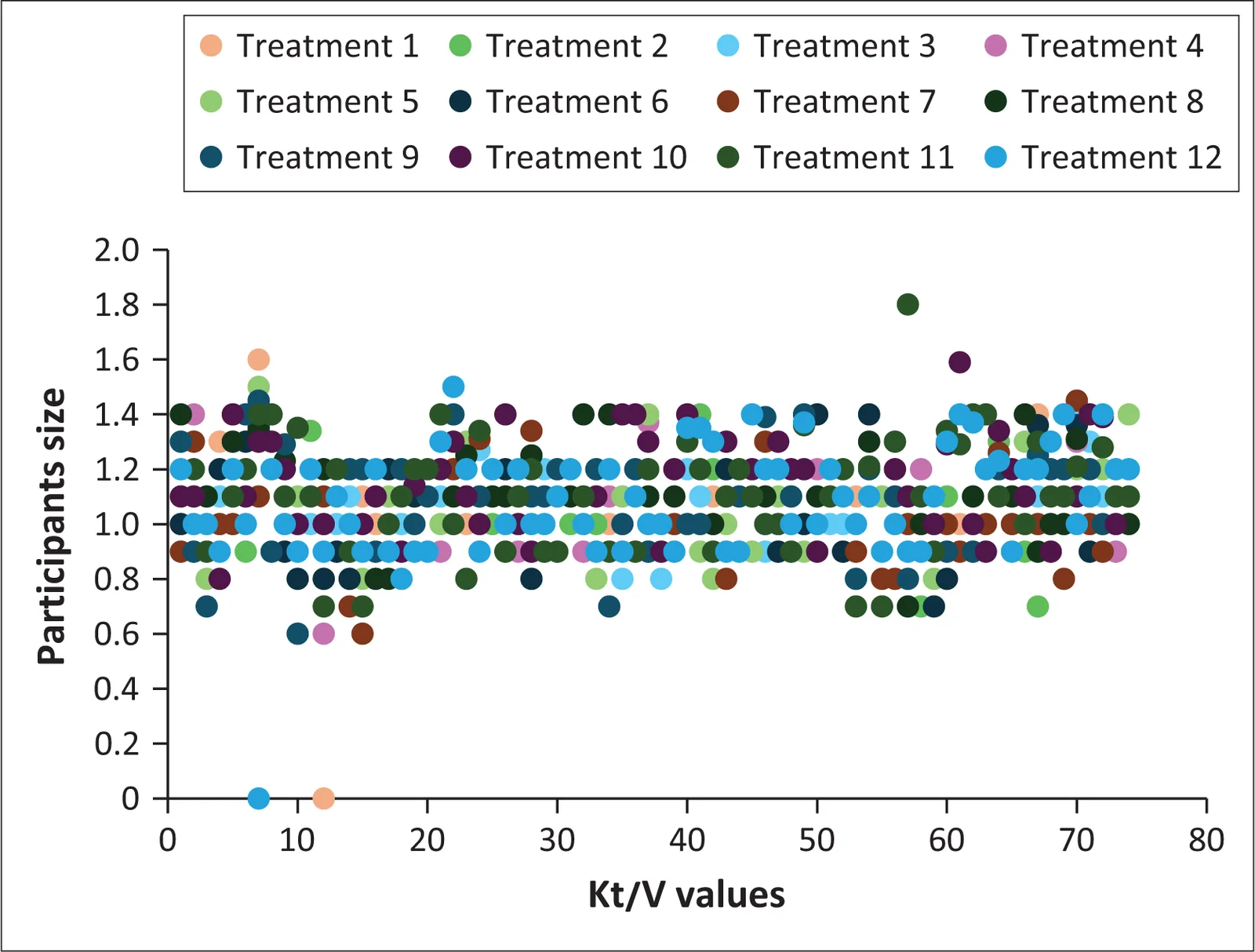
Month 3 daily Kt/V readings.

Figure 1, representing of month 3 from 01 August 2024 to 30 August 2024, demonstrates that the first treatment engaged four respondents, the second treatment involved nine respondents, and the concluding third treatment comprised six respondents who successfully reached the Kt/V target of 1.4.

Creatinine and urea clearance levels were assessed monthly from routinely collected blood samples to further measure dialysis adequacy. Of the 78 respondents, 75 participated in urea testing and 76 in creatinine testing. The outcomes of the clearance assessment are presented in Table 2. It presents detailed descriptive statistics for urea and creatinine, two critical markers of dialysis effectiveness.

**TABLE 2:** The median test for urea and creatinine.

Variables	Urea						Creatinine				
	*N*	Median	25th Pctl	75th Pctl	Min	Max	*N*	Mean	s.d.	Min	Max
Month 1	75	19.40	16.20	22.40	10.20	40.00	76	**791.84**	301.15	239.00	**1504.00**
Month 2	75	20.50	16.50	23.20	10.20	**41.30**	76	769.14	313.64	167.00	1491.00
Month 3	75	19.70	16.50	22.40	10.20	39.20	76	773.91	315.97	**122.00**	**1448.00**
Overall	75	**20.13**	**16.77**	**22.40**	10.20	**39.73**	76	**778.30**	**300.20**	247.00	**14 480**

Note: Bold-faceted values indicate significant value.

s.d., standard deviation; *N*, sample number; Pctl, interquartile range percentile; Min, minimum; Max, maximum.

By comparing central tendency and variability, the Table 2 helps assess how well high-flux haemodialysis is clearing metabolic waste, thereby serving as an indirect indicator of treatment performance.

The median urea level over 3 months was 20.13 mmol/L, with an interquartile range of 16.77 mmol/L – 22.40 mmol/L.

A maximum urea level of 41.30 mmol/L was recorded in month 2, surpassing the overall maximum of 39.73 mmol/L, as presented in Table 2. Month 1 recorded the highest mean creatinine value of 791.84μmol/L, along with a peak of 1504.00 μmol/L, surpassing all subsequent months. The lowest creatinine level, 122.00 μmol/L, was noted in month 3. Additionally, month 3 and the ‘overall creatinine’ category both exhibited a maximum creatinine value of 1448.00 μmol/L. The overall creatinine’s mean was determined to be 778.30 μmol/L, with a standard deviation of 300.20 μmol/L.

### Depression assessment

The respondents completed the BDI questionnaire at the start of the study. The questionnaire was administered once and analysed in consultation with a qualified psychologist. It is a self-report tool completed by every respondent privately at the dialysis centre. The questionnaire was completed anonymously; however, each respondent was assigned a unique identification number for tracking purposes. The questionnaire was distributed to the respondents and collected on the same day once they indicated that they had finished. A few respondents requested to take the questionnaire home to complete and return it at their next dialysis session. The confidentiality declaration was signed by a psychologist who is part of the research team. The BDI questionnaire was utilised to evaluate the severity of depression. Respondents rated their agreement with the 21 items on a 4-point Likert scale, where scores ranged from 0 (‘Never’) to 3 (‘Always’). Based on their scores, the respondents were classified into categories defined by the BDI scoring system. Individuals are classified into six categories, with the first, scores under 10, labelled as ‘Considered Normal’, and the last ‘Extreme’, according to BDI criteria.

The BDI uses a straightforward scoring system:

- 1–10: Considered normal.
- 11–16: Mild mood disturbance.
- 17–20: Borderline clinical depression.
- 21–30: Moderate depression.
- 31–40: Severe depression.
- 40+ Extreme depression.

### Beck Depression Inventory item analysis

Table 3 shows the frequency and percentage distribution of responses to individual BDI items. It highlights the prevalence and severity of depressive symptoms among respondents, providing insight into the psychosocial burden associated with chronic dialysis and its potential interaction with treatment adequacy. Respondents rated 21 items on a 4-point Likert scale ranging from 0 (‘Never’) to 3 (‘Always’).

**TABLE 3:** Beck Depression Inventory item analysis.

BDI questions	Never		Sometimes		A lot		Always	
	Freq	%	Freq	%	Freq	%	Freq	%
Q1. I am so sad and unhappy that I cannot stand	40	**51.28**	27	34.62	9	11.54	2	2.56
Q2. I feel the future is hopeless and that things cannot improve	45	**57.69**	22	28.21	7	8.97	4	5.13
Q3. I feel I am a complete failure as a person	53	**67.95**	18	23.08	6	7.69	1	1.28
Q4. I am dissatisfied or bored with everything	11	14.10	47	**60.26**	18	23.08	2	2.56
Q5. I feel guilty all the time	46	**58.97**	20	25.64	9	11.54	3	3.85
Q6. I feel I am being punished	52	**66.67**	16	20.51	4	5.13	6	7.69
Q7. I hate myself	51	**65.38**	21	26.92	3	3.85	3	3.85
Q8. I blame myself for everything bad that happens	37	**47.44**	28	35.90	8	10.26	5	6.41
Q9. I would kill myself if I had thechance	61	**78.21**	12	15.38	4	5.13	1	1.28
Q10. I cry all the time now	33	**42.31**	29	37.18	2	2.56	14	17.95
Q11. I feel irritated all the time	16	20.51	33	**42.31**	27	34.62	2	2.56
Q12. I have lost all of my interest in other people	23	29.49	33	**42.31**	22	28.21	0	0.00
Q13. I cannot make decisions anymore	25	32.05	29	**37.18**	21	26.92	3	3.85
Q14. I believe that I look ugly	36	**46.15**	24	30.77	15	19.23	3	3.85
Q15. I cannot work at all	10	12.82	30	38.46	34	**43.59**	4	5.13
Q16. I do not sleep as well as I used to	8	10.26	38	**48.72**	27	34.62	5	6.41
Q17. I am too tired to do anything	11	14.10	48	**61.54**	18	23.08	1	1.28
Q18. I have no appetite at all anymore	24	30.77	46	**58.97**	7	8.97	1	1.28
Q19. I have lost more than 15 kgs	29	37.18	39	**50.00**	9	11.54	1	1.28
Q20. I am so worried about my physical problems that I cannot think of anything else	22	28.21	44	**56.41**	10	12.82	2	2.56
Q21. I have completely lost interest in sex	20	25.64	40	**51.28**	13	16.67	5	6.41

Note: Bold-faceted indicate significant values.

BDI, Beck Depression Inventory; Freq, frequency; %, percentage.

Total BDI scores were calculated by summing individual item responses, and the frequency distribution for each item was analysed.

### Beck Depression Inventory categorisation

The median BDI score was 17 (range: 1–50), as presented in Table 4. A median BDI score of 17 indicates that the majority of respondents exhibited depressive symptoms. According to the BDI scoring system, a total score between 17 and 20 falls within the range for borderline clinical depression. Table 4 summarises overall depression severity using total BDI scores. The distribution of scores enables assessment of central tendency and spread, helping to determine the overall mental health status of the study population and its potential relationship with clinical outcomes.

**TABLE 4:** Beck Depression Inventory total score analysis (*N* = 78).

Median	25th Pctl	75th Pctl	Min	Max
17.00	13.00	22.00	1.00	50.00

Note: Analysis variable: BDI total scores.

BDI, Beck Depression Inventory; Pctl, interquartile range percentile; Min, minimum; Max, maximum.

Table 5 classifies respondents into standard BDI severity categories and shows their frequency distribution. It is important because it translates raw scores into clinically meaningful groupings, enabling easier interpretation of depression prevalence and facilitating comparisons with measures of dialysis adequacy. Respondents were categorised based on total BDI scores, and the categories were composed as follows:

- 17.95% were classified as ‘No depression’.
- 82.04% of respondents, according to their scores, are experiencing depression. The levels of depression are detailed as follows:
  ■ Mild: 25.64%
  ■ Borderline: 25.64%
  ■ Moderate: 21.79%
  ■ Severe: 6.41%
  ■ Extreme: 2.56%

**TABLE 5:** The frequency of depressive symptoms.

BDI category	Frequency	%	Cumulative	
			Frequency	%
1. Considered normal	14	17.95	14	17.95
2. Mild	20	25.64	34	43.59
3. Borderline	20	25.64	54	69.23
4. Moderate	17	21.79	71	91.03
5. Severe	5	6.41	76	97.44
6. Extreme	2	2.56	78	100

BDI, Beck Depression Inventory.

### Correlation analysis

Table 6 presents Spearman’s correlation coefficients assessing the relationships between depression scores and clinical variables. It is crucial for identifying potential associations between psychological well-being and dialysis effectiveness, especially where both physiological and psychosocial factors may interact. The statistical analysis was conducted using the Spearman’s correlation coefficient. For this purpose, the BDI total score and overall and Kt/V average are presented in Table 6.

**TABLE 6:** Correlation procedure.

Three variables	BDI total score		
	*r*	*p*	*n*
Overall, Kt/V average	-0.25711	**0.0231**	78
Overall urea	-0.03293	**0.7791**	75
Overall creatinine	0.05224	**0.6541**	76

Note: Spearman’s correlation coefficients; Prob > |r| under H0: Rho = 0; Number of observations. Bold-faceted indicate significant values.

BDI, Beck Depression Inventory; *r*, Spearman’s correlation coefficient; *p, p*-value; *n*, sample number.

A significant negative correlation was observed between Kt/V and BDI scores (*r* = −0.257, *p* = 0.0231), indicating that lower Kt/V values are associated with higher depression levels. Since *r*^2^ = 0.0661, only 6.61% of the change in BDI score is due to the change in overall Kt/V, where 93.39% of the change in BDI score is because of other factors. The BDI and overall urea: The analysis showed a non-significant correlation, with *p* = 0.779, *r* = −0.0329, and *r*^2^ = 0.0011. The BDI and overall creatinine: A non-significant correlation was observed between the two variables, *p* = 0.654 and *r* = 0.0522, with *r*^2^ = 0.0027. A weak but statistically significant inverse relationship was observed between Kt/V and depression scores, indicating that lower dialysis adequacy was associated with higher levels of depressive symptoms. No significant associations were found between depression and urea or creatinine levels.

### Correlation analysis between Kt/V results and Beck Depression Inventory variables

The correlation analysis between the overall Kt/V and BDI total scores is reported in Table 7. It helps determine whether psychological status is associated with differences in treatment effectiveness, which may suggest clinically relevant links between mental health and dialysis outcomes. The categories of mild mood disturbance and borderline clinical depression each comprised 20 respondents. Both the mild mood disturbance and moderate depression categories reached identical maximum Kt/V values of 1.34 and the same median value of 1.03; however, their interquartile ranges differ.

**TABLE 7:** Correlation analysis: overall Kt/V average and Beck Depression Inventory scores.

BDI category	*n* Obs	*n*	Median	25th Pctl	75th Pctl	Min	Max
1. Considered normal	14	14	1.09	1.03	1.11	0.97	1.18
2. Mild mood disturbance	20	20	1.03	0.99	1.11	0.97	1.34
3. Borderline clinical depression	20	20	1.05	1.02	1.10	0.94	1.20
4. Moderate depression	17	17	1.03	1.00	1.07	0.76	1.34
5. Severe depression	5	5	0.98	0.96	1.04	0.96	1.09
6. Extreme depression	2	2	0.99	0.96	1.02	0.96	1.02

Note: Analysis variable: Overall, Kt/V average.

BDI, Beck Depression Inventory; Min, minimum; Max, maximum; Pctl, interquartile range percentile; N, sample number; Obs, observations.

### Reliability analysis

Table 8 reports Cronbach’s alpha (α) values, indicating the internal consistency of the BDI used in the study. Demonstrating strong reliability supports confidence in the measurement of depression and strengthens the validity of subsequent analyses. The BDI demonstrated good internal consistency as shown in Table 8, with a Cronbach’s α of 0.886, confirming the reliability of the instrument.

**TABLE 8:** Reliability test.

Variables	Alpha
Raw	0.884455
**Standardised**	**0.886049**

Note: Cronbach’s coefficient alpha.

## Discussion

The investigation of the correlation between chronic haemodialysis patients’ depression scores and dialysis adequacy among CKD patients in South Africa revealed that CKD brings about several difficult adjustments in patients’ lives. The discussion focuses on three main domains: dialysis adequacy, depression prevalence, and the observed correlation between these variables.

A minimum single-pool Kt/V (spKt/V) of 1.2 per session, with an optimal target of 1.4 for patients on thrice-weekly haemodialysis, is recommended as per the international guidelines (Kidney Disease: Improving Global Outcomes [KDIGO] 2024). In this cohort, only 22 respondents (28.2%) had a mean OCM score > 1.4, while the majority (71.8%) had scores between 1.2 and 1.4. Session-level analysis revealed intermittent attainment of target Kt/V values, suggesting that variability in adequacy may be influenced by modifiable factors such as missed sessions, shortened treatment times, or vascular access issues. These results align with those reported by Breitsameter, Figueiredo and Kochhann (2012), who assessed Kt/V ratios using various calculation methods in a cohort of 159 haemodialysis patients. Their study demonstrated that the average Kt/V measured via OCM was consistently lower than values calculated using the Daugirdas formula. These differences underscore the importance of accurate and consistent adequacy assessment methods. Achieving adequate dialysis is critical, as it directly improves patient outcomes by reducing complications associated with ESKD and enhancing overall quality of life (Jia et al. 2025).

A further investigation consistent with our findings was conducted by Yaseri, Fayazi, and Khatibani (2023), which demonstrated that the average urea reduction rate was 63% ± 10.4% and that optimal dialysis adequacy was achieved in 45.9% of cases. This discovery indicates that an individual’s biographical traits may influence the effectiveness of dialysis, beyond mere assessment by dialysis machines. A research study by Somji, Ruggajo, and Moledina (2020) revealed that the average Kt/V was 1.1, suggesting that patients are not reaching the haemodialysis treatment goals. However, a noteworthy discovery made by Agaba et al. (2003) indicated that approximately one-third 34.3% and 40.6% of patients achieved the target Kt/V (> 1.2), respectively. Proper and optimal dialysis can avert complications, reduce frequent hospital admissions and associated costs, and enhance patient’s quality of life. It is recognised that hemodialysers with a smaller surface area provide less adequate haemodialysis than those with a larger surface area. While inadequate dialysis may contribute to depression, it is also possible that depressed individuals are less likely to adhere to prescribed dialysis regimens, thereby lowering delivered adequacy (Somji et al. 2020).

Our research indicates a median BDI score of 17, with 82.04% of respondents exhibiting different degrees of depression, which aligns with the results reported by Bahall et al. (2023), who found that 62.1% of CKD patients experienced clinical depression. Similarly, Sameeha et al. (2023) identified a depression prevalence of 92.4% and an anxiety prevalence of 83.3% among patients undergoing haemodialysis. Moreover, CKD patients undergoing dialysis were found to have elevated risks of depression and anxiety, along with diminished quality of life. Depression in haemodialysis patients is influenced by factors such as socioeconomic status, employment challenges, dietary restrictions, and non-adherence to treatment protocols (Halili et al. 2021). According to the study conducted by Najafi et al. (2016), depression and anxiety were common in haemodialysis patients, with prevalence rates of 31.5% and 41.7%, respectively. However, their findings indicated that women experienced a high prevalence due to the demands of fulfilling various social roles, which could contribute to heightened anxiety among women. Our findings concerning the prevalence of depression among CKD patients are consistent with those of Ngema and Ramalepa (2025). They further reported the clinical implications of the results, suggesting the need for substantial modifications in the clinical management of kidney failure across Africa. This is compounded by the findings of Butt et al. (2022), who found individuals experiencing kidney failure to be at a heightened risk of developing mental health problems due to the long-term impacts of their illness. They further found that the health-related aspects of mental health warrant particular focus within the growing population of patients suffering from kidney failure.

Patients with higher depressive symptomatology may be less adherent to dialysis treatment, resulting in inadequate dialysis and subsequently contributing to worsening physical and psychological symptoms and poorer overall outcomes (Weisbord et al. 2014). Hung et al. (2011) reported a similarly weak but significant inverse correlation (*r* = –0.2), whereas Klaric and Klaric (2012) found this relationship to be more pronounced in peritoneal dialysis patients. Xi et al. (2016) linked poor adequacy and nutritional status to increased depression risk, supporting the need for integrated nutritional and mental health support in dialysis care. These findings suggest a bidirectional relationship between dialysis adequacy and depression.

Our results are consistent with those of Xi et al. (2016). They found that the risk of depression increases with poorer dialysis adequacy and nutritional status. Thus, improving dialysis adequacy and nutritional status can reduce the incidence of depression. However, a significant correlation could not be drawn for anxiety.

Additionally, Noee et al. (2020) found the adequacy of dialysis to demonstrate a significant correlation with well-being, anxiety, and depression (*p* < 0.001). Additionally, Abdelmobdy et al. (2022) found a statistically significant correlation between BDI severity and access (*p* = 0.001).

In the research conducted by Najafi et al. (2016), no significant correlation was found between the adequacy of dialysis and the occurrence of depression and anxiety among patients. The study evaluated the two measures of urea reduction and Kt/V, revealing that neither of these indicators demonstrated a significant relationship with the prevalence of depression and anxiety in patients.

### Limitations of the study

This study was geographically limited to the Northern Cape and Free State provinces, which may affect generalisability. The final sample of 78 respondents, below the intended target, may reduce statistical power.

Dialysis adequacy was assessed solely via OCM, without validation against alternative methods or consideration of residual renal function. Important clinical parameters, such as body mass index (BMI), haematocrit, ultrafiltration volume, and laboratory-based urea kinetics, were not captured, potentially limiting the depth and interpretability of findings. Future studies should incorporate broader populations, larger sample sizes, and multimodal adequacy measures to enhance validity and applicability. Furthermore, all haemodialysis machines utilised are of the same brand and model; thus, it is recommended to evaluate other machines to ensure the reliability of the results. Online measurement is used as a single marker of adequacy; renal residual function is not considered, pathology is calculated not recorded, BMI, hematocrit and ultrafiltration not included.

Another limitation of this study is that depression was assessed using the BDI tool without a formal clinical diagnostic interview. The BDI measures depressive symptom severity rather than providing a clinical diagnosis of depression, which may introduce misclassification bias. Consequently, some respondents may have been incorrectly classified regarding depression status, which should be considered when interpreting the findings.

A limitation of this study is the limited consideration of the cultural and linguistic validity of the BDI in the South African context. Although widely validated, its use in multilingual and diverse populations may be affected by language interpretation and literacy levels. Respondents with language, literacy, or visual difficulties were assisted by the principal investigator and nursing staff through verbal translation and clarification in English and relevant local languages, thereby improving accessibility but potentially introducing response bias.

## Conclusion

Chronic kidney disease and dialysis have an impact on patients beyond the physical symptoms of the disease.

The results of the current study indicated a significant correlation between the average Kt/V and the total BDI score. This finding suggests that intensive haemodialysis, which entails significant lifestyle changes and expectations, has an impact on patients’ mental health. It is therefore recommended that increased awareness be directed towards the psychological well-being of patients living with CKD. This study identifies interventions such as cognitive-behavioural therapy, structured exercise programmes and counselling as effective approaches to reducing depressive symptoms and enhancing overall quality of life. Incorporating depression screening into the assessment of all pre-dialysis CKD patients is essential. Nephrologists can advise on the appropriate referral of patients in the acute stage to mental health specialists. Additionally, achieving the target Kt/V rate not only improves the clinical outcomes of patients undergoing haemodialysis but may also positively influence their mental health by reducing treatment-related complications, enhancing overall well-being, and potentially alleviating psychological distress associated with inadequate dialysis.
